# Intact FGF23 and Markers of Iron Homeostasis, Inflammation, and Bone Mineral Metabolism in Acute Pediatric Infections

**DOI:** 10.3390/biology13090728

**Published:** 2024-09-17

**Authors:** Eleni Papastergiou, Dimitrios Rallis, Afroditi Papagianni, Vasileios Cholevas, Nikolaos Katzilakis, Ekaterini Siomou, Eftichia Stiakaki, Alexandros Makis

**Affiliations:** 1 Department of Pediatrics, University Hospital of Ioannina, Faculty of Medicine, School of Health Sciences, University of Ioannina, 45110 Ioannina, Greece; 2 Department of Pediatric Hematology-Oncology, University Hospital of Heraklion, Postgraduate Program “Hematology-Oncology in Childhood and Adolescence” of Medical School, University of Crete, 71003 Heraklion, Greece; 3 Neonatal Intensive Care Unit, University Hospital of Ioannina, Faculty of Medicine, School of Health Sciences, University of Ioannina, 45110 Ioannina, Greece; 4 Laboratory of Child Health, Faculty of Medicine, School of Health Sciences, University of Ioannina, 45110 Ioannina, Greece

**Keywords:** acute infection, anemia of inflammation, functional iron deficiency, hepcidin, i-FGF23

## Abstract

**Simple Summary:**

Intact Fibroblast Growth Factor 23 (i-FGF23), a phosphaturic hormone that contributes to the anemia of inflammation, especially in chronic kidney disease, has not been studied extensively in acute inflammatory diseases in pediatric patients with no comorbidity. The aim of this prospective clinical study is to evaluate the association of i-FGF23 with hepcidin and markers of iron homeostasis, erythropoiesis, inflammation, and bone mineral metabolism in acute pediatric infections. As hepcidin levels increase, i-FGF23 levels decrease, making them significantly negatively interrelated. Contrary to hepcidin, i-FGF23 was positively associated with iron metabolism parameters and negatively associated with the duration of fever and markers of inflammation. Further research is required to understand the role of FGF23 in the hepcidin–ferroportin axis in acute inflammation.

**Abstract:**

We intend to evaluate the association of intact Fibroblast Growth Factor 23 (i-FGF23), a phosphaturic hormone that contributes to anemia of inflammation, with markers of iron homeostasis, inflammation, and bone mineral metabolism in acute pediatric infections. Seventy-nine children, aged 1 month–13 years, out of which forty-two were males and thirty-seven females, participated in this study. Children with diseases and nutrient deficiencies causing anemia were excluded. Twenty-six patients had bacterial infections, twenty-six had viral infections, and twenty-seven children served as healthy controls. Complete blood count, markers of inflammation, iron and mineral metabolism, serum hepcidin, and i-FGF23 were compared between the groups. Thirty-nine percent of patients with bacterial infection and twelve percent of patients with viral infection presented characteristics of anemia of inflammation (*p* < 0.001). Ninety-two percent of patients with bacterial infection and eighty-one percent of patients with viral infection had functional iron deficiency (*p* < 0.001). Hepcidin was significantly positively correlated with the duration of fever, markers of inflammation, and negatively with iron, mineral metabolism parameters, and i-FGF23. i-FGF23 was positively correlated with iron metabolism parameters and negatively with the duration of fever, markers of inflammation, and hepcidin. Hepcidin levels increase, whereas i-FGF23 levels decrease in acute pediatric infections. Further research is required to understand the role of FGF23 in the hepcidin–ferroportin axis and for hepcidin in the diagnosis of bacterial infections and mineral metabolism.

## 1. Introduction

Anemia is known to occur in acute or chronic inflammation, including infectious diseases. Hepcidin, the master regulator of iron metabolism, contributes to hypoferremia during inflammation, a state known as functional iron deficiency (FID), by reducing the amount of membranous ferroportin, the protein exporter of iron from cells. Subsequently, iron-restricted erythropoiesis may result in anemia of inflammation. Proinflammatory cytokines, particularly interleukins (ILs), IL-6, IL-1β, and Tumor Necrosis Factor-α (TNF-α), play a role in this by inducing hepcidin expression during inflammation [1,2].

Moreover, significant research has been conducted on the role of Fibroblast Growth Factor 23 (FGF23) in the pathophysiologic axis of anemia and inflammation, especially in chronic kidney disease (CKD). FGF23 is a primary phosphaturic hormone, contributing to the homeostasis of mineral metabolism. It is encountered in circulation either as a full-length peptide, known as intact FGF23 (i-FGF23), or as proteolytic fragments, namely c-terminus FGF23 (c-FGF23) and n-terminus FGF23. Of these, only i-FGF23 is considered biologically active [3,4]. Acute inflammation increases the transcription of FGF23 in experimental models and then triggers the proteolysis of the FGF23 peptide by protease furin. This causes the circulating levels of c-FGF23 to increase, but the levels of i-FGF23 remain unaffected. This proteolytic cleavage decreases gradually in chronic inflammation, resulting in higher circulating levels of i-FGF23 [5,6,7,8]. Human recombinant FGF23 contributes to the hepcidin–ferroportin axis in a dose-dependent manner, with low concentrations increasing while high concentrations of FGF23 decreasing hepcidin levels, as observed in experimental models with hepatocytes [9]. On the other hand, the c-FGF23 peptide seemed to alleviate hypoferremia in a septic mouse model, probably by acting as an endogenous inhibitor of the bioactive i-FGF23 in their common receptors, which led to a reduction of hepcidin levels [10].

The aim of this study was to assess the interrelationship of i-FGF23 and hepcidin during acute inflammation in clinical conditions. Our initial hypothesis was that i-FGF23 promotes hepcidin expression during acute infection, leading to hypoferremia and subsequently anemia in pediatric patients without comorbidities. We also examined the correlation of i-FGF23 and hepcidin with the markers of inflammation and bone mineral metabolism.

## 2. Materials and Methods

### 2.1. Study Subjects

Over an eight-month period, from October 2021 to May 2022, we prospectively recruited children with acute infections who were admitted to the Pediatric Department of the University Hospital of Ioannina, Greece. This study protocol was approved by the institutional review board of the medical center, and written informed consent was obtained from the parents of all the participants. The study protocol was designed to include children ranging in age from 1 month to 16 years. The study population consisted of three groups: (1) children with suspected or proven bacterial infection, (2) children with suspected or proven viral infection, and (3) healthy children who visited the outpatient clinic for routine examination or vaccination and served as controls.

Bacterial infection was defined based on the patients’ clinical symptoms, physical examination, laboratory investigation, and in some cases, on microbiological proof of causative pathogens through cultures, rapid antigen testing, polymerase chain reaction, and serological antibody testing of appropriate biological specimens. For bacterial infections that were not proven microbiologically, we administered antibiotics to the patients according to the criteria of systemic inflammatory response syndrome (SIRS), particularly 2 of the following: (1) fever above 38.5 °C for at least 72 h or core temperature <36 °C, (2) white blood cell count (WBC) >12,000/μL and/or >10% immature neutrophils, (3) tachycardia (heart rate > 2 standard deviations [SDs] above the mean for age) or bradycardia (heart rate < 10th percentile for age), in absence of painful stimuli, external vagal stimulus, drugs, or congenital heart disease, (4) tachypnea respiratory rate > 2 SDs above normal for age [11,12]. Additionally, the diagnosis of acute otitis media was confirmed by an otomicroscopic examination performed by an ear, nose, and throat specialist. In addition to SIRS criteria, the diagnosis of bacterial sinusitis was also accompanied by symptoms such as headache and persistent nasal discharge for over 10 days with purulent secretions for the last 3 consecutive days. Similarly, the diagnosis of bacterial pneumonia was enhanced by the presence of lobar or segmental, rather than interstitial, infiltrates on chest X-rays, bearing in mind that this cannot safely differentiate between bacterial and viral infection. The diagnosis of acute appendicitis peritonitis was established according to the Pediatric Appendicitis Score > 6, the Alvarado score > 7 [13], and the histopathologic evaluation of the appendix following surgery. Cases of occult bacteremia pertained to children under 36 months of age with fever above 38 °C without a source, WBC > 15,000/μL, absolute neutrophil count > 10,000/μL, and C-reactive protein (CRP) > 30 mg/L [14].

Viral infections were diagnosed based on the patients’ clinical condition and laboratory findings. In most cases, the microbiological proof was acquired through rapid antigen testing, polymerase chain reaction, and serological antibody testing. Roseola infantum was diagnosed clinically. 

Every patient with a bacterial infection was matched randomly with a patient with a viral infection and a healthy control of the same age, and whenever possible, of the same gender.

Children with the following diseases or conditions causing anemia were excluded from the study: hemolytic or aplastic anemias according to medical history and complete blood cell count (CBC) of the subjects; CKD; chronic liver disease; active rheumatic diseases; neoplasms; hypothyroidism; malabsorption syndromes including gastritis; peptic ulcer; celiac disease; inflammatory bowel disease; intestinal failure; intestinal parasitic infections; recent operation or heavy hemorrhage; veganism; and dietary insufficiencies of iron, folic acid, and vitamin B12. As far as bone metabolism is concerned, children with vitamin D deficiency, defined as 25 hydroxyvitamin D [25(OH)D] < 12 ng/mL [15], were excluded from the study.

### 2.2. Measurements 

Blood samples were obtained from all children within 72 h after admission, before the administration of antimicrobial medications to patients with suspected bacterial infection. CBC, inflammatory markers [CRP, erythrocyte sedimentation rate (ESR), and serum ferritin], markers of bone mineral metabolism [serum total calcium (Ca), serum phosphate (Pi), and serum 25(OH)D], and iron metabolism [serum iron (Fe), total iron binding capacity (TIBC), and transferrin saturation (TS)] were investigated. TS was derived using the following formula: (Fe/TIBC) × 100%. Blood chemistry and CBC were measured by standard laboratory methods. The 25(OH)D concentration was measured by a chemiluminescence microparticle immunoassay (Abbott Analyser i1000 SR, North Chicago, IL, USA). I-FGF 23 was measured using enzyme-linked immunosorbent assay (ELISA) (Ιmmutopics, San Clemente, CA, USA), with an analytical sensitivity of 1.5 pg/mL, an inter-assay variability of 9.1%, and an intra-assay variability of 4.1%. Hepcidin was measured by ELISA [DRG Hepcidin 25 (bioactive) HS ELISA, Springfield, NJ, USA] with an analytical sensitivity of 0.153 ng/mL, an inter-assay variability of 9.5%, and an intra-assay variability of 2.2%. Anemia of inflammation was defined as a reduction in hemoglobin two SDs below the mean, based on age-specific norms; low serum iron; low to normal TIBC; TS < 20%; serum ferritin within or above normal values for age; along with increased hepcidin levels [16]. FID was defined by TS < 20%, with ferritin levels within or above normal values for age [16,17].

### 2.3. Statistical Analysis 

Statistical analysis was performed using SPSS 24.0 (IBM, Chicago, IL, USA). Descriptive statistics were calculated for all variables. The Shapiro–Wilk or Kolmongorov–Smirnov tests were used to evaluate the distribution of data. The quantitative variables were expressed as mean ± SD when normally distributed and as median [interquartile range (IQR)], if not normally distributed. Categorical variables were presented as numbers (n) and percentages (%). Comparison of quantitative variables between the study groups was conducted using either one-way ANOVA Bonferroni post-hoc analysis or Mann–Whitney test, whereas Yates continuity chi-square test (χ2) or Fisher’s exact test were performed for categorical variables. Spearman’s rho correlation coefficient was used to study the relationship between either hepcidin or i-FGF23 and quantitative variables. Multivariate linear regression analysis was performed to examine the correlation between hepcidin and i-FGF23 adjusted for the infection status and CRP. 

A sample size analysis revealed that a corresponding study population of 51 subjects, with 17 children per group, was able to detect a difference of 15% in i-FGF23 values, considering a mean of 11 ± 2 in healthy children [18], with a statistical type I error of 0.05 and statistical power of 80%. A *p*-value < 0.05 was regarded as statistically significant.

## 3. Results

### 3.1. Clinical and Laboratory Data for the Three Groups of Patients

This study included 79 children, 42 males and 37 females, ranging in age from 1 month to 13 years. Twenty-six patients had a bacterial infection, twenty-six had a viral infection, and twenty-seven were healthy controls. The median age for children with bacterial infections, viral infections, and controls was 31.5 months (16–51), 29 months (16–45), and 28 months (15–59), respectively.

The infections of the patients are listed in Table 1. Most cases were mainly upper and lower respiratory tract infections, with gastrointestinal infections coming second. The laboratory data of the three groups (bacterial infections, viral infections, and healthy controls) are shown in Table 2.

In patients with bacterial infections, the duration of fever, WBC, and percentage of neutrophil count (NEUT) were significantly longer and higher, respectively, than in those with viral infections and controls (*p* < 0.05). CRP, ESR, and ferritin were the highest in patients with bacterial infections compared to controls (*p* < 0.001).

Patients with bacterial infections had the lowest mean hemoglobin level (11.6 g/dL) out of the three groups, but statistical significance was reached only in comparison with controls. Thirty-nine percent of patients with bacterial infections and twelve percent of patients with viral infections presented characteristics of anemia of inflammation (*p* < 0.001). Patients with bacterial infections presented the lowest iron metabolism parameters (Fe, TIBC, and TS) compared to those with viral infections and controls. Ninety-two percent of patients with bacterial infections and eighty-one percent of patients with viral infections had FID (*p* < 0.001).

Regarding bone mineral metabolism parameters, the bacterial infections group had slightly lower values of Ca, Pi, and 25(OH)D compared to the other two groups, but statistical significance was reached only in the case of electrolytes between patients with bacterial infection and controls.

Median hepcidin was significantly elevated in both infection groups compared to controls, with the highest value recorded in patients with bacterial infections (56.9 ng/mL, *p* < 0.001), whereas median i-FGF23 was similarly reduced in both infection groups compared to controls (*p* < 0.001).

### 3.2. Correlations of Hepcidin

Hepcidin was significantly positively correlated with the duration of fever, WBC, NEUT, and other inflammatory markers (CRP, ESR, and ferritin) (*p* < 0.001). On the other hand, hepcidin was significantly negatively correlated with iron metabolism parameters Fe (*p* < 0.001), TIBC (*p* = 0.007), and TS (*p* < 0.001) as well as bone mineral metabolism electrolytes Ca (*p* = 0.003) and Pi (*p* = 0.028). A statistically significant negative correlation was observed between hepcidin and i-FGF23 (*p* < 0.001) (Table 3).

### 3.3. Correlations of i-FGF23

i-FGF23 was significantly positively correlated with markers of iron metabolism, Fe (*p* = 0.004) and TS (*p* = 0.003), and significantly negatively correlated with the duration of fever (*p* < 0.001) and inflammatory markers NEUT (*p* = 0.026), CRP (*p* = 0.003), ESR (*p* = 0.01), and ferritin (*p* < 0.001). Significant correlations between i-FGF23 and bone mineral metabolism parameters did not arise. However, a statistically significant negative correlation was observed between i-FGF23 and hepcidin (*p* < 0.001) (Table 4).

### 3.4. Multivariate Linear Regression Analysis

After adjusting for the infectious status and CRP in multivariate linear regression analysis, i-FGF23 was not correlated any more with hepcidin [standardized coefficient −0.037, 95% confidence interval [CI] (−0.13, 0.05), *p* = 0.412], whereas the absence of infection was the only factor strongly correlated with i-FGF23 [standardized coefficient 11.45, 95% CI (1.97, 20.93), *p* = 0.019] (Table 5).

## 4. Discussion

Our findings suggest that hepcidin increases while i-FGF23 decreases significantly in acute pediatric infections and that their fluctuations depend on the presence of infection. However, this inverse interrelationship between i-FGF23 and hepcidin is not sufficient to demonstrate clearly whether i-FGF23 contributes to FID and anemia through the hepcidin molecular pathway in acute pediatric infections. Furthermore, hepcidin was associated positively with the clinical course of patients, markers of inflammation, and negatively with iron metabolism parameters and bone mineral metabolism electrolytes. i-FGF23 was correlated significantly positively with iron metabolism parameters and negatively with the duration of fever and inflammatory markers.

Many experimental models have evaluated the kinetics of FGF23 during inflammation [5,6,19,20,21]. The state of acute inflammation promotes FGF23 transcription and subsequently the cleavage of the full-length peptide, by protease furin, into its biologically inactive fragments, resulting in increased serum c-FGF23 levels and relatively low i-FGF23 levels [5]. Bayer J et al. have shown that polymicrobial sepsis in mice resulted in significantly elevated levels of c-FGF23 in the first six hours of sepsis, with the ratio i-FGF23/c-FGF23 falling by 80%. Thereafter, there was a gradual decline of c-FGF23 levels with a parallel gradual elevation of i-FGF23 levels [19]. Masuda et al. have observed that the injection of furin inhibitors in septic mice significantly elevated i-FGF23 levels, implying that furin inhibitors malfunction during sepsis, which allows for the increased proteolysis of the FGF23 peptide [21]. The reduced levels of i-FGF23 found in our study of patients with acute infections could be explained by the kinetics of FGF23 during acute inflammation. Similar clinical data comes from a study of 17 adult patients with CKD and bacterial sepsis, who presented increased proteolysis of the FGF23 peptide at the peak of the bacterial infection, which was reduced after the resolution of the infection [22].

Hepcidin–ferroportin axis [2] could explain the negative correlations of hepcidin with iron metabolism parameters in our study. Additionally, hepcidin is a type II acute phase reactant [23], and proinflammatory cytokines induce its expression [1]. In our study, both infection groups presented significantly elevated levels of hepcidin compared to controls. Hepcidin was also positively correlated with the duration of fever and inflammatory markers, which is in accordance with other clinical studies [23,24,25]. Fluctuations of hepcidin levels followed the clinical course of patients with bacterial sepsis [25]. Small clinical studies in the pediatric population have attempted to investigate the role of hepcidin as a biomarker for the diagnosis of bacterial infections, but the requested cutoff value has not yet been identified [23,26,27,28].

FGF23 has been indicated to contribute to hepcidin expression during inflammation, resulting in FID and anemia [10]. i-FGF23 has been shown to contribute directly to the hepcidin–ferroportin axis in experimental models [9,29]. Recombinant human FGF23 affected hepcidin expression in a dose-dependent manner in experimental models with hepatocytes, with low concentrations of FGF23 increasing, whereas high concentrations reducing hepcidin transcripts [9]. We demonstrated that low i-FGF23 levels coexist significantly with elevated hepcidin levels in both bacterial and viral acute pediatric infections, which is in accordance with the experimental data [9]. However, we could not investigate the kinetics of the antagonist c-FGF23 peptide, which rises at greater levels than i-FGF23 at the peak of acute inflammation [5,6,19,20,21,22] and seems to alleviate hepcidin-induced hypoferremia [10]. Additionally, several factors during inflammation, including IL-6, Bone Morphogenetic Protein-6, and Hypoxia Inducible Factor-1a, might interact with complexity in hepcidin expression [9]. Therefore, we could not assume for certain that low values of i-FGF23 directly promote hepcidin expression and subsequent FID and anemia in acute infections in clinical practice, which would render i-FGF23 a possible hepcidin agonist. Further investigation could illuminate this hypothesis. The mainstay of treatment for anemia of inflammation still remains the eradication of the underlying infection. Severe anemia (hemoglobin < 9 g/dL) is associated with adverse outcomes and increased mortality in critically ill patients in intensive care units. Newer therapeutic approaches involving the hepcidin–ferroportin axis could reverse severe anemia in such patients in the future, restricting the need for transfusion or parenteral iron administration [1]. On the other hand, hepcidin agonists might prevent severe sepsis with siderophilic bacteria in patients with iron-overload chronic medical conditions [2].

Clinical studies have revealed negative correlations between FGF23 and hemoglobin, Fe, and TS, mostly in adults with CKD [30,31,32,33]; however, such associations remain controversial in the pediatric population [34,35,36]. In the early stages of pediatric CKD, there seems to be no statistically significant correlations between i-FGF23 and iron status parameters [35], whereas a significantly negative interrelationship has been documented in the late stages of pediatric CKD [36]. This is explained by the altered metabolism of FGF23 in the state of chronic inflammation, particularly in CKD. The initially elevated proteolysis of FGF23, which occurs at the initial stage of CKD, gradually decreases, leading to higher circulating levels of i-FGF23. i-FGF23 further promotes the secretion of proinflammatory cytokines, especially IL-6, IL-1β, and TNF-α, and as a result, a vicious cycle between i-FGF23 and cytokines sustains tissue trauma and chronic inflammation [5]. Cytokines are novel upregulators of hepcidin [2,5]. Under this prism, i-FGF23 may contribute indirectly to hepcidin expression and anemia of chronic inflammation through cytokines induction [5]. The associations of i-FGF23 with markers of iron metabolism have never been investigated in patients with acute inflammation and no comorbidity prior to this study. In this study, i-FGF23 was significantly positively correlated for the first time with Fe and TS in acute pediatric infections, whereas the correlations of hepcidin with iron-related parameters were proven to be stronger. The correlations of i-FGF23 with iron metabolism parameters could be explained either by the inverse interrelationship between i-FGF23 and hepcidin during acute inflammation or they could be a chronological coincidental observation between acute phase reactants and reverse phase reactants. 

Clinical data regarding the correlations of FGF23 with markers of inflammation are controversial. Dounousi et al. demonstrated a significantly positive correlation of i-FGF23 with ferritin but not with WBC and CRP in adult patients with CKD and bacterial sepsis [22]. In pediatric patients with CKD, i-FGF23 was not correlated with inflammatory markers [34], whereas in other clinical studies with either adult or pediatric populations, c-FGF23 presented positive correlations [37,38,39]. In our study, we displayed a negative correlation between i-FGF23 and inflammatory markers, which is rather a chronological coexistence owing to increased proteolysis of the FGF23 peptide during acute infection, when inflammatory markers peak. 

There has been some research regarding bone mineral metabolism in the case of chronic infections and autoimmune diseases [40], but to a lesser extent in the state of acute inflammation. Mild hypophosphatemia or hypocalcemia occurs frequently in bacterial infections but is usually self-isolated [41]. In our study, only patients with bacterial infections presented statistically significant slightly reduced serum calcium and phosphate values compared to controls, as well as significantly lower i-FGF23 and higher hepcidin levels. 

i-FGF23 induces phosphaturia by decreasing the membrane levels of the sodium–phosphate cotransporters, NPT2a and NPT2c, in the kidney. It also downregulates calcitriol metabolism, leading to restricted phosphate reabsorption from the intestine [3,4]. Paradoxically, the lower serum Pi levels of the patients with bacterial infections are not in accordance with their low i-FGF23 values. No significant correlations between i-FGF23 and any of the studied bone mineral metabolism parameters were recorded. Similarly, an experimental septic model has not revealed any significant correlations, even on a molecular level, by quantifying the transcripts of the implicated ion channels in the FGF23 pathway in the kidney [19].

The reason for this is probably the fact that bone mineral homeostasis is regulated by other coexisting factors [40,42], which were not investigated in the current study. Proinflammatory cytokines such as TNF-α, IL-1β, IL-6, and cortisol increase osteoclast proliferation and activity while impairing the formation of new bone tissue [40]. They also boost the expression of the Calcium-Sensing Receptor, a member of class C of the G proteins-coupled receptors, which directly induces calciuria and prevents the release of the bone-friendly parathyroid hormone during sepsis [42]. Furthermore, in the acute phase of sepsis, the leading cause of hypophosphatemia is considered to be the redistribution of phosphate across the cell membranes, which is also affected by reduced intake or intestinal absorption due to illness and use of certain drugs [43].

On the other hand, hepcidin has been shown to preserve bone mass in experimental models, mostly by hindering iron absorption from the intestine and thus allowing for greater calcium absorption [44,45,46]. The correlations between hepcidin and phosphate levels are controversial in the bibliography and come from clinical studies of CKD patients. Hepcidin has either been significantly positively associated with increased serum phosphate levels [46] or presented no significant correlations [34]. However, these findings adhere to chronic inflammatory diseases. This study disclosed a significant negative correlation between hepcidin and bone metabolism electrolytes in the case of acute infection. Our results may simply represent two distinct facts occurring simultaneously at the peak of bacterial infections; further research would shed light on the role of hepcidin in mineral metabolism during acute inflammation.

To our knowledge, this is the first clinical study that evaluates the role of i-FGF23 in FID through the hepcidin pathway in pediatric patients with acute infection and no comorbidity. Nevertheless, there are some limitations. This is a monocenter study with a small study population. Microbiological confirmation of infections was not always achieved, and some patients had localized infections. Due to technical reasons, neither the levels of c-FGF23 nor the serial measurements of i-FGF23 could be retrieved for a better comprehension of their kinetics in association with FID. 

## 5. Conclusions

To recapitulate, serum hepcidin increases, whereas i-FGF23 decreases during acute infections in vivo. Further research is necessary in order to demonstrate whether i-FGF23 contributes to FID and anemia through the hepcidin molecular pathway in acute infections in vivo. Research on a larger scale is demanded for the use of hepcidin as a biomarker for the diagnosis of bacterial infections in clinical practice and on its role in bone mineral metabolism in the state of acute inflammation. 

## Figures and Tables

**Table 1:** Types of infections in the studied patients.

Bacterial Infections (N = 26)		Viral Infections (N = 26)	
Pneumonia (1 case by methicillin-resistant *Staphylococcus aureus*)	8	Common cold, viral upper respiratory tract infections †	7
Acute otitis media—otorrhoea	6	Acute gastroenteritis †	7
Urinary tract infections (*Escherichia coli*)	4	Acute bronchiolitis †	6
Occult bacteremia	2	Viral-associated wheeze †	3
Pharyngitis and tonsilitis (*Streptococcus pyogenes*)	1	Acute laryngitis †	2
Mastoiditis (*Streptococcus pneumoniae*)	1	Roseola infantum	1
Sinusitis	1	† denotes pathogens isolated from some viral infections: *Respiratory Syncytial Virus*, *Rhinovirus/Enterovirus*, *Adenovirus*, *Human Metapneumovirus*, *Cytomegalovirus*	
Acute appendicitis peritonitis	1		
Staphylococcal scaled skin syndrome (Methicillin-sensitive *Staphylococcus aureus*)	1		
Non-typhoidal *Salmonella* enteritis	1		

**Table 2:** Laboratory data of the three groups (bacterial infections, viral infections, and healthy controls).

Parameters	Bacterial Infections (N1 = 26)	Viral Infections (N2 = 26)	Controls (N3 = 27)	*p*-Value
Age, months (IQR)	31.5 (16–51)	29 (16–45)	28 (15–59)	p+: 0.957
Gender, male	13 (50%)	17 (65%)	12 (44%)	p: 0.303
Duration of fever, days (IQR)	4 (3–5)	1 (1–4)	0	p+ < 0.001
WBC, number/μL (SD)	16,808 (7313)	12,222 (4176)	9043 (3103)	p1 *: 0.006 p2 ** < 0.001 p3 ***: 0.084
NEUT, % (SD)	68 (13)	54 (23)	37 (15)	p1: 0.027 p2 < 0.001 p3: 0.002
LYMPH, % (SD)	24 (13)	36 (22)	53 (14)	p1: 0.027 p2 < 0.001 p3: 0.002
MONO, % (SD)	8 (3)	8 (3)	6 (2)	p1: 1.000 p2: 0.200 p3: 0.127
Hemoglobin, g/dL (SD)	11.6 (1.5)	12.3 (0.9)	12.7 (1.2)	p1: 0.143 p2: 0.006 p3: 0.754
Hematocrit, % (SD)	35 (4)	37 (3)	38 (3)	p1: 0.103 p2: 0.020 p3: 1.000
Anemia	10 (39)	3 (12)	0 (0)	*p* < 0.001
MCV, fL (SD)	78.2 (3.4)	79.4 (3.3)	79.7 (3)	p1: 0.519 p2: 0.281 p3: 1.000
MCH, pg (SD)	26 (1.8)	26 (1.3)	27 (1.2)	p1: 1.000 p2: 0.122 p3: 0.663
RDW-CV, % (SD)	13.6 (1.3)	13.1 (0.8)	12.9 (0.8)	p1: 0.136 p2: 0.016 p3: 1.000
PLT, number/μL (SD)	371,962 (128529)	343,115 (96598)	323,185 (60,138)	p1: 0.886 p2: 0.228 p3: 1.000
Ca, mg/dL (SD)	9.6 (0.6)	10 (0.5)	10.2 (0.5)	p1: 0.078 p2 < 0.001 p3: 0.153
Pi, mg/dL (SD)	4.5 (0.8)	4.9 (0.6)	5.1 (0.9)	p1: 0.248 p2: 0.012 p3: 0.720
25(OH)D, ng/mL (IQR)	24.8 (21.6–30.1)	29 (20.7–33.3)	27.7 (24.2–33.4)	p+: 0.121
25(OH)D insufficiency	6 (23)	4 (15)	2 (7)	p+: 0.259
CRP, mg/L (IQR)	91 (51–210)	9.5 (4–23)	2 (1–2)	p+ < 0.001
ESR, mm/h (IQR)	42 (15–56)	11.5 (5–24)	4 (2–7)	p+ < 0.001
Fe, μg/dL (IQR)	18 (12–22)	23 (18–48)	73 (61–101)	p+ < 0.001
Ferritin, ng/mL (IQR)	88 (53–182)	57 (38–88)	29 (19–38)	p+ < 0.001
TIBC, μg/dL (IQR)	303 (272–323)	339 (299–376)	342 (313.5–368)	p+: 0.003
TS, %, (IQR)	5.9 (5–9)	7.7 (5.6–14.3)	23.2 (17.2–29.3)	p+ < 0.001
FID	24 (92)	21 (81)	0 (0)	p+ < 0.001
Hepcidin, ng/mL (IQR)	56.9 (32.7–121)	42.1 (18.5–83.5)	9.5 (6.3–12.6)	p+ < 0.001
I-FGF23, pg/mL (IQR)	4.9 (2.2–6.4)	3.3 (1.7–5)	11.8 (7.5–22.7)	p+ < 0.001

Data are expressed as mean (SD), median (IQR), or numbers (percentages). Abbreviations: WBC, White Blood Cell count; NEUT, percentage of neutrophils; LYMPH, percentage of lymphocytes; MONO, percentage of monocytes; MCV, Mean Corpuscular Volume; MCH, Mean Corpuscular Hemoglobin; RDW-CV, Red Cell Distribution Width-Coefficient of Variation; PLT, Platelet count; Ca, serum calcium; Pi, serum phosphate; 25(OH)D, 25 hydroxyvitamin D; CRP, C-Reactive Protein; ESR, Erythrocyte Sedimentation Rate; Fe, serum iron; TIBC, Total Iron Binding Capacity; TS, Transferrin Saturation; FID, Functional Iron Deficiency; I-FGF23, intact Fibroblast Growth Factor 23; p1*: comparison between bacterial and viral infections; p2**: comparison between bacterial infections and controls; p3***: comparison between viral infections and controls; p+: comparison between either bacterial infections or viral infections and controls.

**Table 3:** Correlations between hepcidin and the variables i-FGF23, complete blood count, bone metabolism, markers of inflammation, and iron metabolism.

Hepcidin	r	*p*-Value
i-FGF23	−0.495	<0.001
Days of fever	0.700	<0.001
WBC	0.481	<0.001
NEUT	0.531	<0.001
LYMPH	−0.560	<0.001
MONO	0.306	0.006
Hemoglobin	−0.215	0.056
Ca	−0.335	0.003
Pi	−0.247	0.028
CRP	0.596	<0.001
ESR	0.455	<0.001
Fe	−0.722	<0.001
Ferritin	0.566	<0.001
TIBC	−0.303	0.007
TS	−0.688	<0.001

r denotes correlation coefficient (Spearman rho). Abbreviations: I-FGF23, intact Fibroblast Growth Factor 23; WBC, White Blood Cell count; NEUT, percentage of neutrophils; LYMPH, percentage of lymphocytes; MONO, percentage of monocytes; Ca, serum calcium; Pi, serum phosphate; CRP, C-Reactive Protein; ESR, Erythrocyte Sedimentation Rate; Fe, serum iron; TIBC, Total Iron Binding Capacity; TS, Transferrin Saturation.

**Table 4:** Correlations between hepcidin and the variables i-FGF23, complete blood count, bone metabolism, markers of inflammation, and iron metabolism.

I-FGF23	r	*p*-Value
Hepcidin	−0.495	<0.001
Days of fever	−0.427	<0.001
WBC	−0.162	0.155
NEUT	−0.250	0.026
LYMPH	0.255	0.023
MONO	−0.120	0.293
Hemoglobin	0.081	0.478
Ca	0.153	0.178
Pi	0.204	0.072
CRP	−0.327	0.003
ESR	−0.288	0.010
Fe	0.323	0.004
Ferritin	−0.365	0.001
TIBC	0.079	0.490
TS	0.328	0.003

r denotes correlation coefficient (Spearman rho). Abbreviations: I-FGF23, intact Fibroblast Growth Factor 23; WBC, White Blood Cell count; NEUT, percentage of neutrophils; LYMPH, percentage of lymphocytes; MONO, percentage of monocytes; Ca, serum calcium; Pi, serum phosphate; CRP, C-Reactive Protein; ESR, Erythrocyte Sedimentation Rate; Fe, serum iron; TIBC, Total Iron Binding Capacity; TS, Transferrin Saturation.

**Table 5:** Multivariate linear regression analysis with dependent variable i-FGF23 and independent variable hepcidin, adjusted for the absence of infection, and CRP.

Variables	Standardized Coefficient	95% CI	*p*-Value
Absence of infection	11.450	[1.97, 20.93]	0.019
CRP	0.017	[−0.06, 0.1]	0.668
Hepcidin	−0.037	[−0.13, 0.05]	0.412

Abbreviations: i-FGF23, intact Fibroblast Growth Factor 23; CRP, C-Reactive Protein; CI, Confidence Interval.

## Data Availability

The dataset is not publicly available due to the hospital privacy policy.
